# Shaeer’s Cavernotome for Penile Prosthesis Implantation in Scarred Corpora Cavernosa

**DOI:** 10.5152/tud.2023.22189

**Published:** 2023-03-01

**Authors:** Osama Shaeer, Kamal Shaeer

**Affiliations:** Kasr Alainy Faculty of Medicine, Cairo University, Egypt

**Keywords:** penile implant infection, priapism, penile prosthesis infection, cavernotome, corporal scarring, corporal fibrosis, corer

## Abstract

**Objective:**

Penile prosthesis implantation in scarred corporal bodies is one of the most challenging urologic procedures, with high risks of perforation and/or failure. We present Shaeer’s Cavernotome (patent application number PCT/EG2021/050003). This is the forward-cutting cavernotome that relies on the principle of controlled coring and grinding rather than forward stabbing, with fibrous tissue accommodated into the hollow core, thereby ensuring high efficacy and low risk of perforation.

**Materials and methods:**

This is a prospective study involving 18 patients with severe corporal scarring. Surgery is performed through a peno-scrotal incision with an indwelling urethral catheter. Corporotomies are incised and a 2-cm-long core of fibrous tissue is excised with a scalpel. Shaeer’s Cavernotome is introduced and lodged against the fibrous tissue. Coring proceeds with the stretched corpus or crus held between the thumb and index fingers of the non-dominant hand as a guide, ahead of the tip. Shaeer’s cavernotome doubles as a sizer. Following coring, penile prosthesis implantation proceeds.

**Results:**

Dilation of the corpora cavernosa was successful in 17 out of 18 patients. Average coring time was 8 ± 3.2 minutes. Dilation was up to girth 13 Hegar in 12 patients, and 11 in 5. No perforations or infections were encountered.

**Conclusion:**

Shaeer’s cavernotome facilitates penile prosthesis implantation in scarred corporal bodies. Full excavation of both corpora cavernosa is achievable in less than 10 minutes, with a low risk for perforation.

**Main Points**

- Cavernous fibrosis (cavernous scarring) occurs due to neglected priapism or following the removal of an infected penile implant. Implantation of a penile prosthesis in such cases is one of the most difficult challenges in prosthetic urology.
- Existing cavernotomes can scrape off fibrous tissue, provided a track is established. Establishing a track involves forcibly pushing surgical instruments against resistance which frequently ends in perforation or urethral injury, not to mention the failure of establishing the track.
- Shaeer’s cavernotome is a corer that grinds through and cores fibrous tissue by slowly advancing rotatory movements, thereby excavating fibrous tissue more effectively and safely. The cavernotome serves both purposes, establishing the track and widening it, by one pass.

## Introduction

Penile prosthesis implantation in scarred corporal bodies is one of the most challenging urologic procedures.^1^ Scarred corporal bodies are the result of recurrent or neglected ischemic priapism, the removal of an infected penile implant, among other rare causes such as cavernositis. Fibrous tissue obliterates the corpora cavernosa rendering their dilatation both difficult and risky. It is not uncommon that dilation of the scarred corporal bodies will end up in distal perforation, proximal perforation, or urethral injury. The extended operative time increases the risk of infection. Some cases will end up with the failure of dilation. Abandoned cases with several failed surgeries exist worldwide.

Several surgical techniques have been developed to prevent corporal scarring in the first place,^2,3^ others to facilitate opening a track through the scar tissue for implanting the prosthesis.^1,4-7^ Furthermore, several instruments have been devised to make dilation easier and less risky, including the cavernotomes and reverse-cutting scissors.^1,8,9^

**Figure 1.**
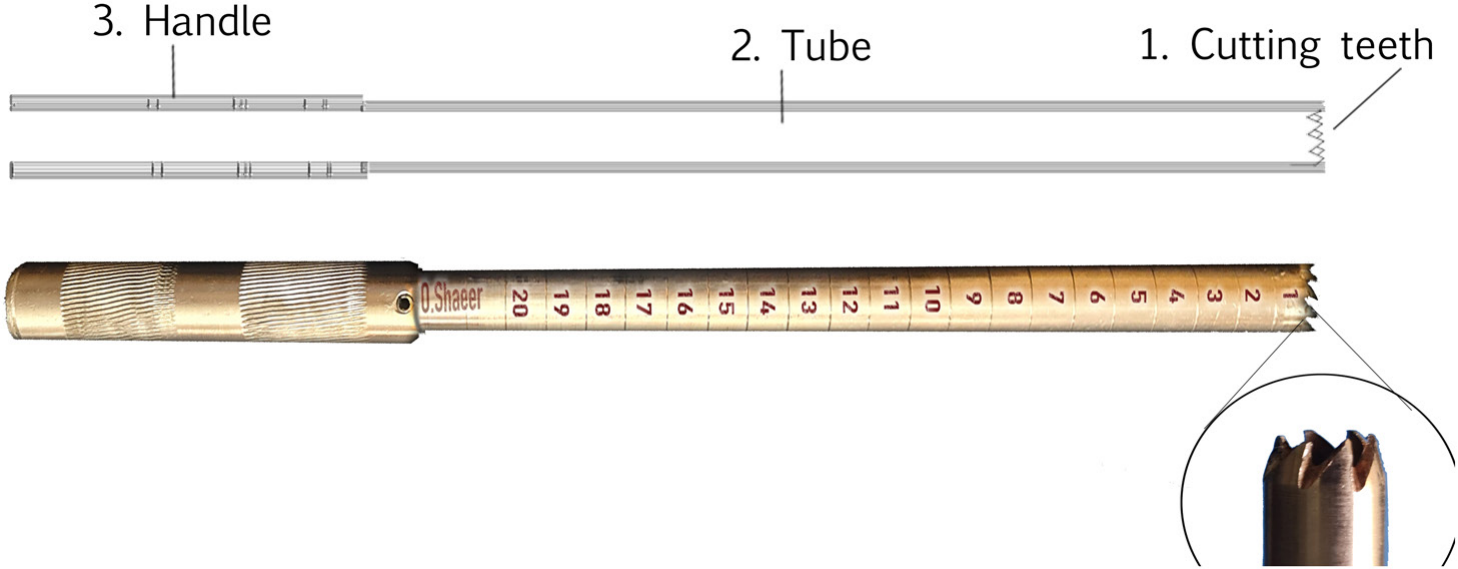
Shaeer’s cavernotome design.

In this work, we present Shaeer’s cavernotome (ShC) (Figure 1). This is the forward-cutting cavernotome that relies on the principle of coring and grinding rather than forward stabbing, the first of a kind. The involved coring movement is controlled, slowly advancing, with minimal force, thereby decreasing the risk of perforation. Coring and grinding effectively demolish fibrous tissue into the hollow core, thereby decreasing resistance to advancement through corporal scarring.

Shaeer’s cavernotome is registered under patent application number PCT/EG2021/050003. It is formed of a hollow tube, with the cutting edge on one end and a handle on the other end (Figure 1). The cutting edge is in the form of 1-mm-thick triangular blades arranged around the perimeter of the tip. The blades point sideways rather than forward to further decrease the risk of perforation. The hollow handle can be either fixed or detachable. The tube is calibrated along its length in 1-cm increments so that progress into the corpora and crura can be measured and so that it can double as a sizer. Shaeer’s cavernotome is available in different girth measurements (6-13 Hegar). Variants include a reusable metal format and a single-use format that is under development.

## Materials and Methods

A total of 18 patients with corporal scarring were operated upon using ShC. Most patients were referred to our tertiary care center from other centers (n = 16) following an announcement. We also recalled 2 patients of our own.

Twelve patients had a penile implant which was removed on account of infection. The range of delay post explantation was 4 months to 60 months (mean 12 ± 4.2). Nine of those patients had several implantations and removals on account of re-infection; twice in 6 and thrice in 3. Those patients were deemed by their respective surgeons as inoperable.

Six patients were referred with neglected post-priapism scarring, 5 being sickle cell anemia patients. The average duration since the last attack was 7 ± 2.6 months. One patient suffered priapism post intra-cavernous injection of papaverine with failed evacuation and shunt surgery, finally accepting the implant and referred, 5 months after the last intervention. Written informed consent was obtained from all patients. Ethical approval # AND-CAI-REC3002, Date 20/1/2021 was obtained from the ethical committee of the Department of Andrology, Cairo University, Egypt.

### Surgical Technique

Surgery is performed through a peno-scrotal incision with an indwelling urethral catheter. The scrotal septum is brought down to expose the crura. Corporotomies are incised, 2-5 cm long, according to the implant intended; whether inflatable or malleable. A core of fibrous tissue is sharply excised with a scissor or scalpel, approximately 2 cm long, right underneath the corporotomy. This is to make space for the introduction of ShC, as well as to test the maximum girth of ShC that will fit in. Selecting that maximum girth of ShC from the start will allow 1-step cavernotomy and dilatation, obviating the need for further excavation.

### Distal Coring

**Figure 2.**
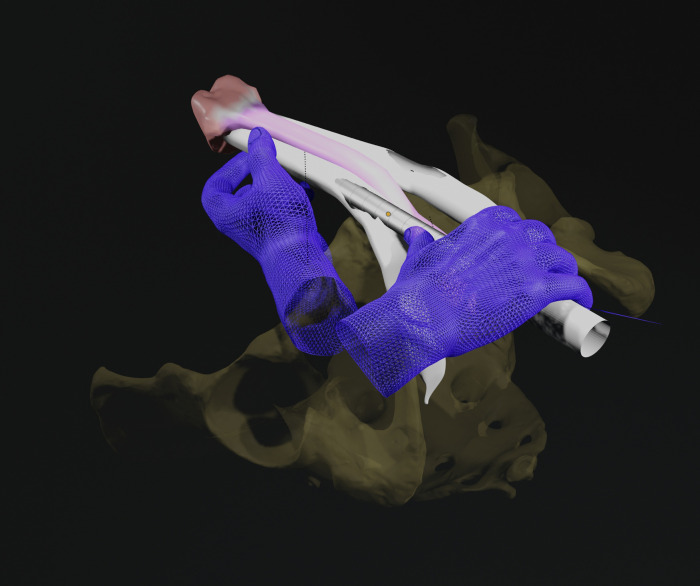
Distal coring—hand orientation.

The distal corpora are cored first. The urethra is identified by the catheter. Shaeer’s cavernotome is introduced through the corporotomy pointing distally and is lodged against the fibrous tissue. The assistant pulls on the stay sutures in the counter-direction to help lodging. The corpus cavernosum is held between the thumb and index fingers of the non-dominant hand right above the tip of ShC, with the intention of checking the position of the tip throughout the process and to stretch and straighten the corpus above the advancing tip. The dominant hand grabs the rear handle. With those preparations in place, coring of fibrous tissue may commence. (Video, Figure 2).

Shc cavernotome is rotated clockwise, while slowly advancing forward in millimeter increments. The non-dominant thumb and index advance ahead of the tip. The direction of coring is central, along the vertical axis, parallel and lateral to the urethra. This is contrary to the customary lateral direction when blunt dilators are used. The non-dominant thumb and index fingers check this orientation at all times. The thumb pushes the spongiosum aside. Coring stops shortly before the tip of the corpus cavernosum.

Coring the distal fibrous tissue takes approximately 1 to 2 minutes per corpus cavernosum. Following coring, the corpora are sized as regards length with the numerical markings on ShC. Blunt dilators can be used to calibrate and maximize girth. The corpora are flushed with an antibiotic solution.

Shaeer’s cavernotome is withdrawn and demolished fibrous tissue is pushed out of it using a straight obturator provided. The same process is repeated on the contralateral side.

Coring leaves behind a thin strand of fibrous tissue that can be pulled out and severed at the tip. However, this is unnecessary, since, in our experience, the strand will not prevent easy insertion of the maximum girth Hegar.

### Proximal Coring

**Figure 3.**
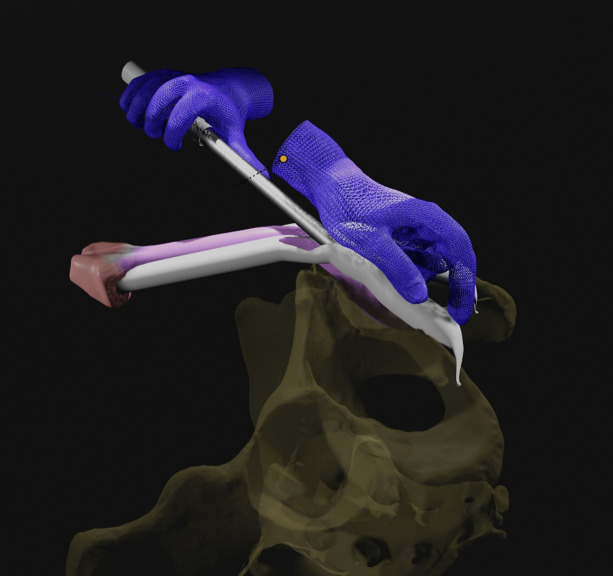
Proximal coring—hand orientation.

Shaeer’s cavernotome is re-introduced into the corporotomy pointing proximally and sideways along the direction of the crus. The non-dominant index or thumb finger is laid over and along the crus to act as a guide for the direction of advancement and to identify the proximal tip/bone (Video, Figure 3). Both can also pinch the crus ahead of ShC’s tip as with distal coring. Coring proceeds as described earlier. Stretching and straightening the crura straight by pulling on the stay sutures or the penile shaft is very important, to avoid side perforation. Advancement stops before the proximal tip which is identified by the non-dominant index finger, as well as by the “bounce-test.” The bouncing test is whereby ShC is repeatedly pushed and pulled gently for a very short distance toward the bone. If it bounces, then coring should proceed further. If it does not or if it bounces for a very short distance, then we have hit-bone and coring is deemed sufficient. Sizing and flushing are performed, followed by coring the contralateral crus. In trained hands, coring each crus takes 1 to 3 minutes.

Following coring, penile prosthesis implantation proceeds in the usual fashion. The follow-up period for the cases presented herein was 14 ± 2.2 months.

## Results

Full dilation of the corpora cavernosa was possible in 17 out of 18 patients. The average coring time was 8 ± 3.2 minutes. Dilation was up to girth 13 Hegar in 12 patients, and girth 11 in 5. In the patient where coring was not possible, the corpora were thinned out and calcified. Size 6 ShC could not be introduced. Corporal reconstruction was performed.

A 3-piece inflatable (regular-size cylinders) was implanted in 3 patients, and a malleable in 15. The choice of the implant was based on cost considerations since penile prosthesis implantation (PPI) is not covered by health insurance

In the 17 patients in whom coring was successful, no perforation, extrusion, or urethral perforation was encountered

The case of corporal reconstruction ended up with infection and explantation. In the cases where coring was successful (n = 17), no infections were encountered through the follow-up period of 14 ± 2.2 months.

## Discussion

There is a continuous evolution of techniques and instruments that tackle the challenge of implantation into scarred corporal bodies. Those techniques and inventions have helped widen the circle of experienced implanters who are able to handle such difficult cases. Prevention of corporal scarring in the first place is now possible with space-preserving casts (Carrion Cast)^3^ and anti-fibrotic agents such as Mitomycin C (Shaeer’s technique),^2^ in addition to appropriately early intervention post priapism or post explantation.

When corporal scarring is already an unfortunate fact, and blunt dilators fail to go through, sharp dissection with scissors or a scalpel can be performed. The reverse-cutting scissors by S.K. Wilson is a simple and effective tool in this regard.^1^ Alternative methods include penoscopy^4,6,7^ and ultrasound-guided excavation.^5^

This step (establishing the track) can be the most difficult and risky in the whole process, with a high possibility of perforation and/or failure. Perforation is due to the forceful stabbing motion against resistance. If establishing the track fails, options include open excavation, corporal reconstruction with graft,^10^ or extracorporeal trans-septal implantation.^11^

Shaeer’s cavernotome is about establishing the track without said forceful stabbing motion. Instead, coring is performed in an incremental slowly advancing fashion, with the blades pointing sideways rather than forward, and the hollow core accommodating the demolished scar tissue. This increases the efficacy of excavation and decreases the risk of perforation. In trained hands, full coring of both corpora cavernosa can be achieved promptly, in less than 10 minutes, lending to a shorter operative time and thereby lowering the risk of infection.

Whatever the method for establishing the track is, once established successfully, it can then be dilated girth-wise with cavernotomes. Those include the Carrion-Rossello cavernotome (Coloplast Corporation, Minneapolis, Minn, USA)^9^ or the Uramix-Mooreville cavernotome (Uramix, Inc. Lansdowne, Pa, USA).^8^ Specialized implants with downsized cylinders can be implanted if needed.

In our experience with ShC, no complementary cavernotomy was required. Most cases were dilated to size 13 Hegar with 1 pass of ShC. The minimum girth post-coring was 11 Hegar, which was proportionate to the overall size of the penis. However, complementary excavation can always be performed by the existing side-cutting cavernotomes, particularly if the initial entry with ShC was with a narrower size than desired. Shaeer’s cavernotome is meant to complement rather than replace the existing cavernotomes.

Despite the low complication rate encountered, all complications of penile prosthesis implantation are possible including perforation, crossing over, and infection. It should be remembered that an instrument with a cutting tip is in use, in a case with resistant fibrous tissue. The procedure should still be reserved for the more experienced and high-volume implanters, despite the presented advancement.

Limitations of the study include the limited sample number. However, the rarity of cases of penile fibrosis should be taken into consideration. Another limitation is the lack of comparison to other existing cavernotomes.

Although excavation using Shaeer’s Cavernotome appears to be effective and relatively safe, some tough situations may call for tough measures. It should be noted that in some cases, further procedures may be needed, such as open corporal excavation, secondary corporotomy incisions, or total corporal reconstruction.

Shaeer’s cavernotome is an instrument that facilitates penile prosthesis implantation in scarred corporal bodies. Cavernotomy and excavation are via non-forceful coring and grinding rather than forceful forward stabbing, hence relatively low risk of perforation. With the full excavation of both corpora cavernosa achievable in approximately 10 minutes, the shorter operative time lends to a lower risk of infection.
